# Constellations of pain: a qualitative study of the complexity of women’s endometriosis-related pain

**DOI:** 10.1177/2049463720961413

**Published:** 2020-10-07

**Authors:** Sarah J Drabble, Jaqui Long, Blessing Alele, Alicia O’Cathain

**Affiliations:** School of Health and Related Research (ScHARR), University of Sheffield, Sheffield, UK

**Keywords:** Endometriosis, chronic pain, pelvic pain, dysmenorrhea, dysuria, dyschezia, dyspareunia, qualitative, interviews

## Abstract

**Introduction:**

Prior research into endometriosis-related pain has focused on specific aspects of the pain experience such as cyclical pain, emotional aspects of pain and certain types of pain such as dysmenorrhea and dyspareunia. However, research has paid less attention to the diversity and complexity of women’s pain experiences, which can lead to failure to recognise some symptoms as part of endometriosis and poor symptom management.

**Methods:**

We conducted qualitative semi-structured face-to-face interviews with 20 women in the United Kingdom recruited from an endometriosis self-help group with a diagnosis of endometriosis via laparoscopy. A topic guide framed questions around experiences of pain. Interviews were audio-recorded and transcribed verbatim. Transcripts were analysed using inductive thematic analysis.

**Results:**

Women experienced multiple types of pain that they felt were caused by endometriosis and affected many different parts of the body including bowel, bladder, lungs, kidneys, nerves, upper body, lower limbs and head. These pains consisted of different conceptual categories: type, pattern and intensity. These categories came together to create a complex, interrelated experience for each individual that we termed ‘constellations of pain’ because each woman had a complex set of pain categories and no two individuals appeared to have the same pain experience.

**Conclusion:**

The complexity and diversity of endometriosis-related pain found in this study has implications for improving diagnosis, medical and non-medical pain management and improving the clinical encounter between women and healthcare professionals.

## Introduction

Endometriosis is a chronic gynaecological condition affecting around 10% of women of reproductive age.^1,2^ Although predominantly depicted as a female disease,^3^ endometriosis has also been found in a minority of men and transgender males.^4 [Bibr bibr4-2049463720961413],5^ It is typified by the presence and growth of endometrial cells outside the uterus^6–8^ and is found most commonly on the surface of the ovary, but also the fallopian tubes, pelvic cavity, abdominal cavity, liver and sometimes the lungs.^9^ Pain is one of the most common symptoms of endometriosis, along with fatigue, abnormal excessive menstrual flow (menorrhagia) and infertility.^9–11^

Endometriosis pain often continues even after treatment^12,13^ and impacts quality of life^14–16^ including affecting identity, social and family life, sexual quality of life,^17–22^ ability to work,^15 [Bibr bibr18-2049463720961413][Bibr bibr19-2049463720961413][Bibr bibr20-2049463720961413][Bibr bibr21-2049463720961413],23^ mental health,^21,24,25^ and causing disability,^16,26 ,27^ which Hallståm and colleagues referred to as *a ruined life*.^28^ Dealing with these consequences and feelings of difference from others mean women struggle to create a sense of coherence across different aspects of their life^28^ which can result in poor mental health including anxiety and depression.^16,29,30^

### Types of pain

The literature on descriptions of endometriosis-related pain has largely focused on measuring pain in order to create outcome measures for clinical research studies rather than for clinical encounters between patients and healthcare professionals.^31^ This literature has three classic presentations of endometriosis-related pain: dysmenorrhea (pain during menstruation), non-menstrual chronic pelvic pain, and dyspareunia (pain during sexual intercourse).^16^ Measures of endometriosis-related pain have focused primarily on cyclical dysmenorrhea and non-menstrual pelvic pain; for example, the Endometriosis Daily Pain Impact diary^32^ and the Endometriosis Pain and Bleeding diary.^33^ More recently, measures have been developed to include other types of pain such as pain on defecation (dyschezia), painful urination (dysuria), ovulation pain, lower back and groin pain, upper body pain including breast pain, upper back and shoulders, headache and migraine.^34–36^ Some of these pain measures have been criticised for lacking patient input, limitations in describing endometriosis accurately,^9,36^ and not reflecting patient’s concerns and priorities.

There have been calls to consider qualitative approaches to pain research^37^ and some qualitative studies have focused on descriptions and experiences of pain. Endometriosis pain has been described as ranging from a minor irritation to being totally overwhelming or paralysing.^10,26,38^ Denny^9^ found that the intensity and duration of pain along with experiencing pain during sexual intercourse (dyspareunia) differentiated endometriosis pain from normal period pain. However, women who have experienced painful periods since adolescence have nothing to compare this experience against^26^ and may normalise endometriosis pain. This normalisation also occurs because experiences and beliefs about endometriosis-related pain and gynaecological pain more generally are gendered, bound up in what women are expected to feel and put up with.^3,26,27^ Women are expected to deal with severe pain within the confines of daily life without complaint, creating discourses of disempowerment^39^ and consequences for those who cannot or will not accept this burden.^26^ Research has also found that women with endometriosis often lack the tools to express the severity of their pain to healthcare professionals without resorting to overused metaphors that may not be believed,^40^ reflecting the difficulty in accurately communicating the lived experience of pain.^41^ In particular, Bullo^40^ found that the complexity of pain experience is difficult to describe because the ways in which pain is described are overused and cannot convey the magnitude and complexity of individual experiences. In addition, issues of uncertainty around aspects of endometriosis such as cause, diagnosis and the best treatment can affect how women and health professionals interpret and manage the pain.^25^ We argue that research around endometriosis-related pain to date has discussed some aspects of women’s pain experiences, such as how pain affects their quality of life and the psychological and emotional aspects of pain, but does not offer detailed descriptions of its diversity and complexity. Understanding more about this may help researchers to understand which interventions might be helpful to women, and provide clinicians with more understanding about how different endometriosis pains are for different women. This study explores the complexity and variability in how women with endometriosis experience their pain.

## Method

### Design

We conducted a qualitative interview study with 20 women diagnosed with endometriosis. We obtained ethical approval from the School of Health and Related Research (ScHARR) Ethics Committee (Study 156019) at the University of Sheffield.

### Sampling and recruitment

We initially approached participants through a local endometriosis support group in the north of England. These local groups exist throughout the United Kingdom, meeting monthly to offer women support in living with and managing their condition. We approached a convenience sample of women attending group meetings and via an online Facebook group and email mailing list. We introduced the study, inviting only those with a laparoscopically confirmed diagnosis of endometriosis to contact the researchers or leave their details if they were interested. Snowball sampling was then used with participants and support group leaders to increase the diversity of the sample by identifying participants with more complex and rare types of endometriosis. These women were then approached directly via email to ask if they were willing to take part. Participants received an information sheet detailing the study. We used email or telephone calls to arrange interviews at a time and place convenient for participants. We approached 20 participants for interview who were all interviewed. This sample gave us rich data with expert participants and was large enough for replication to occur within conceptual categories leading to data saturation.^42^

### Data collection

Interviews took place between May 2017 and August 2018. After obtaining written informed consent, B.A. (a clinician undertaking an MSc dissertation under supervision of S.D., qualitative researcher) conducted 10 interviews following a topic guide developed from the existing literature that included describing symptoms, describing in-depth the different types of pain, and where and how the women experienced their pain. These interviews were analysed for the MSc dissertation. The research was excellent but needed a larger sample for data saturation. J.L. (qualitative researcher) conducted a further 10 interviews looking to extend the diversity of types of endometriosis. Interviews were conducted in participant’s homes (n = 12), private rooms in workplaces (n = 4) and at the university (n = 4) depending on participant preference. As this was a potentially emotive subject, participants were informed that they could stop the interview at any time if they wanted to and were free to withdraw at any point. If they became distressed, they were asked if they required any further help from the support group or other healthcare professionals. All interviewees were able to continue the interview. Interviews lasted between 30 and 105 minutes. Interviews were digitally audio-recorded and transcribed verbatim by a university-based transcription service. We gave all participants pseudonyms to preserve anonymity in the results.

### Analysis

We did a two-stage analysis using NVivo™. B.A. coded the first nine interviews thematically using the six phases outlined by Braun and Clarke^43^: familiarisation with the data, generating codes, searching for themes, reviewing themes, defining themes and writing up the themes. Throughout the process, B.A. discussed themes with S.D. In the second stage of analysis, J.L. coded the remaining 11 interviews to existing themes and identified additional themes in discussion with S.D. In describing the complexity that we were seeing in the data, we brought together the themes of types, patterns and intensities of pain into individual constellations to show how they interrelated to create individually diverse experiences of pain. Although it is not common to count numbers of participants in qualitative research, we chose to display in brackets the numbers of women in our sample who described particular types of pain to show that women experienced more than one type of pain.

## Results

Our participants were 20 UK women over 18 years of age with a laparoscopically confirmed diagnosis of endometriosis. Participants were mainly aged between 31 and 40, employed, and married or in a long-term relationship. Most had experienced symptoms for over 10 years, with eight participants waiting over 10 years for a diagnosis (Table 1). While we did not collect data directly on comorbidities, the women in our sample reported other conditions such as polycystic ovary syndrome (PCOS) (n = 4), fibroids (n = 1), adenomyosis (n = 2), non-endometriosis cysts (n = 1), anaemia (n = 1), irritable bowel syndrome (n = 1), rheumatoid arthritis (n = 1), acephalgic migraine (n = 1) and hypertension (n = 1). All women were taking some form of pain medication. This ranged from over the counter medication such as paracetamol, ibuprofen and low-dose co-codamol, to strong prescription pain killers such as nonsteroidal anti-inflammatory drugs (NSAIDs), opioids, non-opioids, migraine-specific medication, benzodiazepam, anti-depressants and epilepsy medication. Some participants had a cocktail of medications to take when the pain went from mild to severe.

**Table 1.** Participant characteristics (n = 20).

Participant characteristics	N
Age	
21–30	5
31–40	12
41–50	2
>51	1
Employment	
Employed full-time or part-time	15
Employed but off sick due to endo	1
Unemployed/not currently working	4
Relationship status	
Married/long-term relationship	17
Single	3
Ethnic background	
White British	16
White European	2
Black African	1
Missing data	1
Duration of symptoms (years)	
<5	3
5–10	5
>10	12
Time since diagnosis (years)	
<5	14
5–10	4
>10	2
Delay in diagnosis (years)	
<5	5
5–10	7
>10	8

### The complexity of endometriosis pain

**Figure 1.**
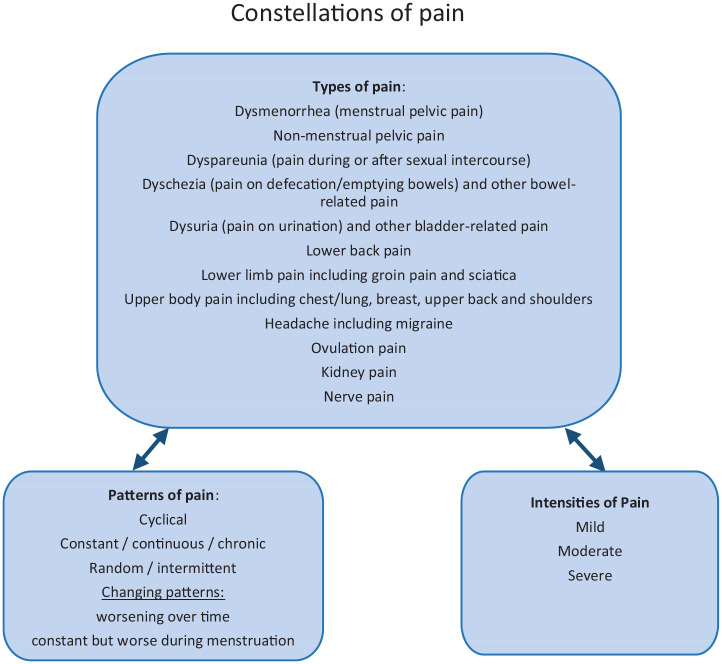
A conceptual understanding of endometriosis-related pain by pain types, patterns and intensities forming constellations of pain.

We first present the conceptual categories identified from participants’ descriptions of their pain based on a wide variety of types of pain, patterns of pain and intensities of pain to create a conceptual understanding of the complexity of pain in endometriosis (Figure 1). We second show how these conceptual categories came together to create a complex, interrelated experience for each woman that we have termed ‘constellations of pain’, highlighting the complexity and uniqueness of each woman’s pain. We illustrate this by drawing complex pain maps of these constellations for two women. We also describe some of the consequences of pain including the psychological and emotional impact.

### Types of pain

There were many different types of pain described by participants depending either on the site of occurrence in the body, for example, groin, nerve, muscle, or its occurrence during a particular activity, for example, during urination or sexual intercourse. Participants reported more than one type of pain, ranging from three to eight, with over five types reported on average.

#### Dysmenorrhoea and non-menstrual pelvic pain

Pelvic pain occurred in the lower abdomen during menstruation (dysmenorrhea, n = 16) and outside of menstruation (non-menstrual pelvic pain, n = 17). These types of pain varied in sensation that was linked to intensity. It was described by some as ‘an ache’ and ‘like a sort of cramp’ which felt ‘like someone’s pushing or squeezing inside’, others described it as a ‘stabbing pain’ almost ‘like somebody’s got a knife on your inside’. For a few others, it was a pain that ‘came in waves’ and felt like their ‘muscles were slowly being ripped out’:

I can’t get a handle on the pain, I can’t sit, I’m not comfortable, like I’m kneeling on the floor and laying with my head on the sofa clutching my belly. . .like somebody’s punched you really hard or something but constantly. (Isabel)

Some had the pain predominantly on one side of their abdomen, while for others it could occur on any side. For some women, non-menstrual pelvic pain would worsen around their period, becoming severe dysmenorrhoea.

#### Dyspareunia (pain during or after sexual intercourse)

Participants described pain related to sexual intercourse (n = 14) as a ‘drawing, dragging pain’ that radiated down their thighs; others talked about experiencing ‘a sharp stabbing-type pain’ during intercourse and sometimes up to a few days afterwards. Some women experienced pain during any form of intercourse while others felt it only after deep penetration or during orgasm, causing them to avoid intercourse completely; this could put a strain on relationships and affect self-esteem:

Sometimes it can be ok and it’s not until orgasm that its painful and I can be just curled up in a little ball because it’s really uncomfortable, which is obviously not very nice when it’s supposed to be a pleasurable thing. (Fiona)

There were also differences in the pattern of how women experienced dyspareunia; some only felt it around menstruation, while for others it represented a constant problem throughout the month.

#### Dyschezia (pain on emptying one’s bowel) and other bowel-related pain

With the onset of endometriosis, opening up the bowels became ‘really painful’ for some participants (n = 15), with one describing it as ‘a pin was being shoved into your bowel’ (Yvonne). For some participants the pain would sometimes start long before they needed to use the toilet. Dyschezia was sometimes also associated with bloating and a change in bowel habit to either diarrhoea or constipation or a combination:

I was going between constipated and diarrhoea quite frequently throughout the week and all in a day sometimes. And it almost felt like, even with diarrhoea it felt like that sort of constipated sharp, almost like a tearing inside feeling. . . (Yvonne)

The pain could be more severe or occur only around menstruation:

emptying my bowels can be quite painful. It’s strange because it’s only painful when the rest of my pain is going on. It’s not like it’s an ongoing thing throughout the month. (Fiona)

Some participants found that constipation aggravated the pain and could be a side effect of pain medication, which for one participant felt like a bowel obstruction. Another found that the cause of her bowel pain was the attachment of her bowels to her ovaries causing ‘excruciating’ pain.

#### Dysuria (pain on urination) and other bladder-related pain

Some participants (n = 10) described dysuria and other bladder-related pain. Participants often described dysuria as a ‘burning pain’ on urination similar to experiencing a urinary tract infection. This was usually worse in the mornings, especially if their bladder was already full and needed emptying, and sometimes intense enough to wake them up:

And also the more full my bladder was the more painful it was on emptying and for a while afterwards. (Olivia)

Participants also noticed that concentrated urine could serve as a pain trigger:

if I didn’t have a lot of water before I went to sleep I was guaranteed to have the pain in the morning, so was I just sensitive to like how concentrated my urine was. (Yvonne)

Increasing fluid intake to circumvent concentration of urine was also problematic however, as it could result in higher frequency of urination that could be painful and frequent trips to the bathroom during the night, affecting sleep.

Of those participants who did not experience dysuria, one reported having had their bladder repositioned during surgery for endometriosis on the bowel and kidneys, while another had difficulties starting to urinate and felt like she was

weeing over a bubble [] it feels like there is something there. (Naomi)

#### Low back and lower limb pain

Fewer participants (n = 5) reported having ‘constant’ back pain in addition to other symptoms. The pain was present throughout their menstrual cycle but could be particularly bad during menstruation:

I developed this horrible back pain along my lower back that is now persistent with me and particularly during periods. (Susanne)

Pelvic pain was often reported as radiating down to the lower limbs (n = 12), causing limited mobility when it occurred. This could sometimes be sudden, and was described as ‘sciatica-type pain’ (Fiona):

I could be walking on the street and would just get it and I’d just have to stand still because you can’t even move. There’s nothing else that you can do. (Val)

#### Other types of pain

Some women experienced other types of pain that they linked to endometriosis due to other symptoms occurring at a similar time. Upper body pain was most commonly reported (n = 9), which if severe could lead to breathlessness. This included chest pain that one participant reported came from a collapsed lung, and breast, back and upper body pain. A similar number described headaches or migraines (n = 8) that could occur in the run up to or during menstruation, and could be accompanied by other symptoms such as sensitivity to light and noise and numbness. A few reported ovulation pain (n = 3), which was cyclical and occurring mid-cycle when ovulation was due to take place. It was described as being a ‘dull sensation’ by one participant, while another said she felt her ovaries were being pinched:

I can feel my ovaries and it feels as if someone’s got a pair of pliers that you would separate electric wires with. It feels as if someone’s pinching the ovaries like that. (Barbara)

However, for women attempting to conceive, ovulation pain helped to inform them when they were ovulating and from which ovary, although doctors had told them it was impossible to know when or on which side they ovulated.

Other less common pains were kidney pain (n = 3), earache (n = 1), muscle and joint pain (n = 1) and severe nerve pain (n = 2):

I was getting stabbing, really sharp stabbing pains, like somebody was sticking a needle in my eye like jabbing it in my eye, then I’d, you know 2 seconds later it would happen in my knee, [oh, ok] or the bottom of my foot, or it would shoot out of my toe nail, my hands were and my skin was burning in different places, the soles of my feet were burning. (Mel)

### Patterns of pain

Participants described four different patterns of pain – cyclical, constant, random and changing.

#### Cyclical pain

Most women noticed a pattern to their pain, experiencing cyclical dysmenorrhea, dyspareunia or constant pain that became worse around menstruation:

It seems to end within a week or sometimes two, and then I will find I’m spending the next few weeks drained, and getting sort of a vicious circle. (Nicola)

Some women described how dyschezia, dysuria, ovulation or migraines also had a cyclical pattern. Some women felt dyspareunia only around the time of their period, while for others it represented a constant problem throughout the month.

However, cycles could vary from a couple of weeks to a few months depending on hormonal medication and other conditions such as polycystic ovaries. The pain also often lasted longer than menstruation or built up for several days before but eased as menstruation started. Others described the pain worsening during menstruation and often accompanied by heavy bleeding, or continuing beyond the end of their period.

#### Constant pain

Some participants reported experiencing pain on a daily basis rather than varying during their monthly cycle:

I can’t remember the last day when there wasn’t something. (Sally)

The pain could be ‘constant’, ‘switched on’ and persistent although varying in intensity:

. . .and then for whatever reason. . .something just switched on and from that moment on its been a constant like period-like pelvic pain, and it would hurt, for a few days it would hurt like a lot. (Yvonne)

#### Random or intermittent pain

Although some women noticed a fairly predictable pattern forming around their monthly cycle, others reported no consistency in their pain:

My pain is very very strange, sometimes like people would get it during the time of their period, mine is just random, it comes whenever it wants to. . . I never knew when it was coming. (Bethan)

Participants also described certain triggers of pain such as foods and drinks, stress, or not drinking enough. Participants perceived the ‘random’ nature of their pain as ‘strange’ and unpredictable, which was challenging because there was no way to prepare for something that could happen anywhere and anytime. Participants described particular difficulties dealing with this pattern; for example, one participant was dreading her wedding day in case she had a flare up while another described not knowing how to prepare for trips away in case she needed hot water bottles and how much pain medication to take.

#### Changing patterns of pain

Participants also described how the pattern or intensity of their pain changed over time. For some participants, pain worsened through the day, while for others there was a progressive worsening in severity over a period of months or years, changing from only happening during menstruation to being a constant throughout the month. Constant pain could still vary in intensity, with some participants noticing triggers such as opening the bowels, or cyclical patterns as described above:

Everything was happening you know 24/7 throughout the month, but certainly there would be a peak before my period and during my period and then also towards the end of my period. (Mel)

Pain could reduce after medical treatment or surgery only to worsen again resulting in worsening quality of life and the need for stronger medication; in one case, pain even increased after surgery.

### Intensities of pain

Pain went from mild pain, where participants felt able to function, to more moderate pain and severe, disabling pain. Mild pain was described as ‘uncomfortable’, ‘dull’, ‘annoying’ pain, around 3 or 4 out of 10, that could be ignored more easily or managed with over-the-counter painkillers such as paracetamol or ibuprofen and did not interfere with quality of life. Moderate pain was described as ‘quite strong pain’, ‘really painful’ ‘bent over double’ ‘between a 7 and an 8’. Participants described how it could significantly affect daily life, but could be managed by stronger painkillers. Some women however experienced such severe pain that it was disabling despite taking large amounts of strong painkillers. Participants described being bedridden, scoring the pain as 10 out of 10, and reducing them to tears, experiencing nausea, vomiting, fainting or even causing a seizure.

. . .and I’d be crawling to the toilet on all fours because if you [] wake up in the morning or even in the middle of the night and you sit up and swing your legs out of bed, I’d just be gone, I’d just faint. I was that weak and in that much pain. (Barbara)

The pain became so unbearable for some that they attended emergency departments; for example, one participant described having difficulty breathing but struggled to receive appropriate help, sometimes being misdiagnosed with appendicitis or ectopic pregnancy.

It was not always the case that some participants had mild pain while others had severe, as for some participants, severity varied throughout their cycle:

green is like my symptoms are so mild they don’t bother me or are non-existent, orange is they bother me and they interfere with what I’m doing but I can still do stuff, and red is I can’t do anything, it’s completely wiped me bedridden. (Helen)

Another aspect of pain linked to intensity was how invasive the pain was, that is, how much the pain interfered with everyday life. While severe pain was often more invasive, this was not always the case. For example, one participant described how it was easier to ignore a dull moderate pain than another milder but more constant pain:

it can be mild but it can still be very insistent and distracting, whereas the dull sensation I can kind of put in the corner of my brain and ignore. (Helen)

In contrast, other participants described how they could ignore more constant pain because they had become accustomed to it, and just pushed through it:

just like a dull ache, I suppose like period pain, but it’s there all the time [] which I’ve grown to, it’s just there I know it’s there. (Hannah)

### Constellations of pain

**Figure 2.**
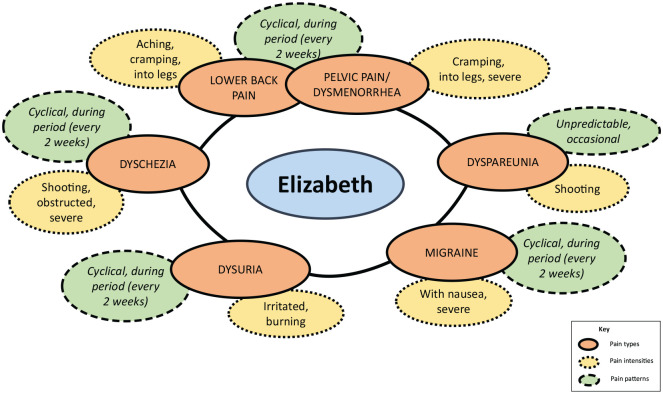
Elizabeth’s constellations of pain.

**Figure 3.**
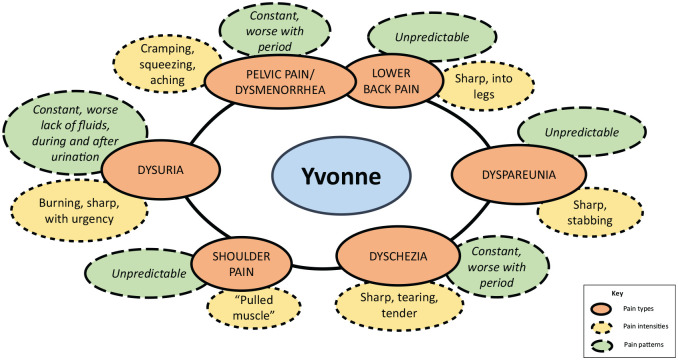
Yvonne’s constellations of pain.

The different aspects of pain described above did not exist in isolation, but were interrelated into constellations of pain experienced by individual women. Figures 2 and 3 show constellations of pain for two participants in which pain type, pattern and intensity interrelate in different configurations; so each type of pain has its own pattern and intensity. In Figure 2, Elizabeth described six different types of pain including five types of cyclical pain (dyschezia, dysuria, dysmenorrhea, migraine and lower back pain) and one type of random or intermittent pain (dyspareunia). She experienced pain affecting her pelvis, bowel (severe shooting pain), bladder (irritation and burning), lower back and legs, (aching and cramping) and head (severe migraines). It impacted on bodily functions such as urination, bowel movements (bowel feels obstructed) and sexual intercourse (shooting pain) and was accompanied by other symptoms such as nausea. Although the pain was cyclical, Elizabeth’s cycle was extremely short so the pain was every 2 weeks rather than the expected 4 weeks.

In Figure 3, Yvonne also described five similar types of pain (dyschezia, dysuria, dysmenorrhea, migraine and lower back pain). Unlike Elizabeth, however, Yvonne also described shoulder pain rather than migraine. The pattern of pain for Yvonne was also different, with three types of constant rather than cyclical pain (pelvic pain worsening to dysmenorrhea, dyschezia worsening during menstruation and dysuria worsening around urination or from a lack of fluids). While she shared Elizabeth’s random dyspareunia, Yvonne also described both her back pain and shoulder pain as random and intermittent rather than cyclical. While there were some similarities in the descriptions of some pain (lower back pain), there were notable differences. Yvonne’s description of dysuria matched Elizabeth’s to some extent (burning) but she also described urgency and sharp pain. Dyschezia seemed qualitatively different with Elizabeth describing it as shooting and severe, while Yvonne described it as sharp, tearing and tender. Yvonne also described a cramping, squeezing and aching pain in the pelvic area, which Elizabeth described as severe cramping into her legs.

### Consequences of pains and other related symptoms

While pain was a significant aspect all the participant’s endometriosis experience, the majority also reported many other related symptoms caused by or related to the pain. Fatigue (n = 15) was frequently a direct result of pain, but could also be due to anaemia from heavy bleeding, broken sleep from dealing with pain or other symptoms such as frequent urination, or medication that caused tiredness or insomnia. Women often reported that pain was bound up with psychological and emotional symptoms stemming from living with pain, which caused low mood, mood swings or anxiety (n = 12), which caused pain to be more intense or harder to tolerate when they were anxious or down. Many medications also affected mood, particularly hormone treatments which for some triggered severe mood swings or depression which some found harder to manage than the pain. Other effects of endometriosis and/or medication, such as diagnosis delays, weight gain, social isolation and relationship or work difficulties also significantly influenced self-esteem and mood:

I’ve not always been bent over double in pain, I’m probably in pain in my brain, where I’ve been tired, stressed tired, when your eyes are heavy, and you can’t cope and it is, it’s horrible you feel like you are by yourself, it is a depressive disease to be in. (Wendy)

A number of women (n = 9) experienced anxiety linked to difficulties conceiving, or uncertainty as to whether they would struggle to conceive in the future. Heavy bleeding was experienced by 12 participants, sometimes accompanied by clots, long periods and/or frequent periods (i.e. more often than once a month). Bloating was also a common problem (n = 9), sometimes causing significant discomfort that could be cyclical or constant and made some participants need to wear bigger clothes or look pregnant, particularly difficult if they were unable to conceive. Poor concentration and memory problems (n = 7) were also difficult to deal with, along with a range of digestive symptoms in the bowel (n = 5), and nausea and/or vomiting (n = 4) that was accompanied by dizziness for two participants. Other symptoms included hot sweats (n = 3), skin problems related to medication or repeated use of hot water bottles (n = 3), low-grade fever (n = 2), weight gain (n = 2) and nosebleeds (n = 1).

## Discussion

In this article, we have presented three conceptual categories relating to endometriosis-related pain: pain types, patterns and intensities. We showed a wide range of variation within each of these categories and then how they interrelated to create what we termed constellations of pain that are unique to each woman’s pain experience. Our findings have implications for research and clinical practice.

### Conceptual understanding of endometriosis-related pain

Research into endometriosis-related pain often focuses on cyclical dysmenorrhea and non-menstrual pelvic pain and how they impact quality of life and more recently mental health.^14,16,30^ We found examples of the three classic forms of endometriosis pain identified by Bourdel et al.^44^: dysmenorrhea, non-menstrual chronic pelvic pain and deep dyspareunia. Like other researchers we also found evidence of many other types of endometriosis-related pain, including dyspareunia,^15,34,36,45^ chronic and cyclical dyschezia, dysuria, ovulation pain, lower back pain, headache including migraine and groin pain.^15,34,36^ Our research also adds to the limited evidence around endometriosis affecting the upper body, including breast, upper back and shoulder pain.^36^ In addition to types of pain already identified in the literature, we found that women also linked earache, muscle and joint pain, and nerve pain to their endometriosis. These other types of pain have received less attention because they are not related to surgical diagnosis^46^; however, our research has shown that they are still problematic for the women experiencing them.

Endometriosis-related pain is often presented as cyclical pelvic pain occurring in the run-up to, or during menstruation. Some research has challenged this representation, for example, in the previous works,^26^,^36^ and our study supports that. We found that some women in our sample experienced random episodes of pain that they found particularly challenging to deal with because it created unpredictability leading to challenges dealing with uncertainty^26^,^28^ and creating coherence in life.^28^ We also found that some women experienced constant pain that increased in intensity during menstruation and for others pain started as cyclical but worsened to be more severe,^33^ persistent and pervasive, which has been linked to poor quality of life.^47^ Research has suggested that experiencing pain may lead to sensitisation of the nervous system^12,13,48^ recognised as a reason for pain to become more pervasive over time^49^ however, participants believed that their endometriosis was worsening.

In addition, we found that intensity did not always relate to how intrusive pain was to everyday life.^48^ Some less intense pain was experienced as more difficult to manage. Research has suggested that these differences in attentional focus are important in understanding how pain affects lives because severe pain can consume every experience^50^ or prevent attention being paid to other aspects of experience leading to suffering.^51^

### Strengths and limitations

We used semi-structured interviews to understand how women with endometriosis experienced their pain. Most endometriosis-related pain research we found was aimed at creating clinical measures of pain for clinical studies, and as such represents the product of a particular epistemological community.^41^ In contrast, our study aimed to describe the complexity and diversity of endometriosis-related pain from the perspective of the women experiencing it. The women we recruited were from a local endometriosis support group and may not be representative of all women with endometriosis, in terms of pain experience and socio-demographic characteristics such as ethnic diversity. Women with severe pain may have been more likely to agree to be interviewed than women with more minor symptoms. We also did not collect details of comorbidities or lists of pain medications although we asked women about these. However, a strength of our study is the representation of the complexity of the different types, patterns and intensities of pain, and how individuals experience these constellations, as this is not well represented in the literature. Future research should consider the experiences of minority groups including men and transgender males who may not identify with these descriptions.

### Clinical implications of findings

Participants’ experiences of endometriosis-related pain were variable, complex and embedded in wider experiences of endometriosis symptoms. Research has highlighted the importance of understanding ‘pain as a lived event’^51^ in which it is essential to understand how the person understands their pain and how it impacts on their life. Difficulties in describing experiences of pain due to its invisibility and subjective nature can lead to a vagueness in describing symptoms,^9,40^ while the gendered nature of endometriosis-related pain can mean severe pain is normalised.^3,26,27^ These difficulties mean that patients may not be believed or find it difficult to communicate the severity of their pain without resorting to cliches.^40^ Furthermore, if clinicians^39^,^40^ focus on presentation of cyclical pain as a diagnostic factor,^46^,^52^ women who present with other experiences, such as random or constant pain may be disbelieved adding to delays in diagnosis and treatment and feelings of frustration and disempowerment.^39^ Issues of uncertainty around aspects of endometriosis such as cause, diagnosis and the best treatment can affect how women and health professionals interpret and manage the pain^26^ and creating issues for diagnosing endometriosis through presentation of cyclical pain.

In this article, we have identified a number of types, patterns and intensities which clinicians could use to help women describe the multiplicity and complexity of their pain experiences, which women may not have associated with endometriosis, or may have feared would be dismissed as hysterical or exaggerated.^3,27,41^ The complexity of the constellations of pain presented in this study has implications for the psycho-emotional impact of pain on quality of life, which suggests that clinicians need to take a wider biopsychosocial approach^53^ to reflect the full lived experiences of pain in people with endometriosis. We suggest that Figure 1 could act as a tool to allow women to describe their constellation of pain to clinicians.

## Conclusion

We have shown that women with endometriosis can experience a complex array of pain symptoms that are not only cyclical but can be constant or random, creating difficulties with unpredictability. The descriptions of pain displayed here could help people with endometriosis and clinicians communicate about pain types, patterns and intensities, which might help with earlier diagnosis and identifying strategies for symptom relief.
